# Dysentery, (So Called) Entero Colitis

**Published:** 1888-07

**Authors:** J. S. Todd

**Affiliations:** Atlanta, Ga., President Medical Association of Georgia, Professor Materia Medica and Therapeutics, Atlanta Medical College, etc., etc.


### DYSENTERY, (SO-CALLED.) ENTERO-COLITIS.*

By J. S. TODD, M. D., ATLANTA, GA.,

President Medical Association of Georgia, Professor Materia Medica and Therapeutics, Atlanta Medical College, etc., etc.

Errors in diagnosis between dysentery and enteritis are very liable to occur, especially during epidemics of the former. I am persuaded that such a mistake is often made. I confess to having been so unfortunate myself on several occasions. Indeed, while the text-books make the difference so plain that it would seem that a blunder was impossible, nevertheless, mistakes occur. Even diarrhœa is often mistaken for dysentery, and *vice versa*. Says Da Costa, after differentiating between them as only he can, "yet in practice we meet with cases which commence with diarrhœa and end in dysentery, or begin with dysenteric symp-

*Read before Atlanta Society of Medicine, June 19th, 1888.

toms and terminate in diarrhœa, and in which it becomes, therefore, puzzling to say whether we are dealing with the former or latter disorder." Says the same author, "colitis is not always dysentery, and dysentery is often more than mere colitis."

During the summer just passed, and also to a limited extent this spring, a fatal form of dysentery, so-called, was prevalent over all northern and middle Georgia. I am sure many lives were lost by the too prevalent practice of treating disease by name—but more of this *anon.*

The leading symptoms in the great majority, if not all the cases, for the first day or two are clearly dysenteric, the lesion being referable to the rectum and colon. There is at this time, i. e., after 36 to 48 hours, generally no fever, but, on the contrary, the temperature ranges from 97° to 98°, or even lower; tormina and tenesmus disappear; the actions are moderately large, streaked with blood and mucus, very offensive, and are followed by a sense of relief, which is so strikingly different from dysentery simple. The thirst abates notably, the pulse increases in rapidity and loses in strength. Nausea begins to develop, and later on vomiting supervenes and becomes the most troublesome and dangerous symptom. Palpation of the bowels will reveal, as a rule, more tenderness around umbilicus and in right illiac fossa than along the course of colon. Borborygmi and tympanitis are heard over the entire abdomen wherever auscultation and percussion are practiced—the former more especially over small bowels, the latter along transverse and descending colon. The duration of cases that end in recovery is from two weeks to a month. The prognosis grave—for the fatality in many localities was as high as 25 per cent.

Never forgetting that dysentery is really a disease of constipation, a purgative is of course the first thing to be given. I prefer calomel, followed by castor oil. The salines are often efficient, but *frequently* defective. They do not *empty* the bowels as does oil. Watery discharges, often repeated, follow their administration, and still sciballae and other offending substances are are not certainly dislodged. After being sure that the bowels are cleared, then opium. For the tormina and tenesmus of simple dysentery no better adjuvant of opium can be employed than the warm hip bath.

The success of the ipeccuanhua treatment in dysentery is so well attested by competent and honest observers, and sustained by statistics so satisfactory and overwhelming, that to attempt to refute or deny it would be idle.

But when the symptoms I have detailed are present, its employment borders on madness. Still, I have known more than one doctor to keep it up until his patients died ; and happily for the sufferers, the evil day was not long delayed.

Now opium is given, but where there is so much derangement of the digestion, is it absorbed ? The patient does not sleep after its repeated administrations in large dose, *per orem*—opium is a soporific—his actions are as numerous—opium is an astringent—clearly something is wrong. I once prescribed three grains of morphine in three ounces of water, and directed that a teaspoonful be taken after each action. In twelve hours the patient took the entire quantity; in other words, had twenty-four actions during that time. A hypodermic injection of 1-6 gr. of morphine in this case carried the same patient through the next twelve hours without an action on his bowels, he sleeping most of the time. "Medicines, to exert a remote effect on the economy, must be absorbed into blood or internal fluids of the body" is an axiom of Headland, that it would be doubly well for us to consider when treating the disease in question.

For the persistent nausea and vomiting, first of all give the stomach rest by withholding food and medicines. Medicines given to "settle" the stomach do more harm than good as a rule. Get rid of the idea that your patient will die of starvation in a few days. Remember, that food not digested is on a par with medicines not absorbed.

A mixture of one teaspoonful each of turpentine and mustard to a tablespoonful of lard rubbed over bowels, or applied to the face of a poultice, relieves the tympanitis very often, and also the nausea, and it frequently does away with the necessity for a blister. A vessicant is, however, in some cases indispensable, and should cover the entire abdomen; but remember not to blister deeply. Let it remain on only a few hours; watch it closely and remove on the first appearance of goose flesh; put back the poultice; it will cause it to fill. The contraction of the capillaries that is caused by a superficial blister is persistent; whereas, if the vesication be deep, dilatation of them supervenes.

I had a patient last summer who rejected everything given by the mouth, even pledgets of ice—absolutely *everything* for eight days, although she had been blistered—who was saved by hypodermic morphine every six hours.

In a case recently under my care that vomited so persistently, I, morning and night, used morphine and atropine hypodermically, and had laudanum and starch given per rectum after each action. A recovery was made in ten days; during five of these no aliment, water (carbonated) or otherwise was retained; no blister was used.

In each of these cases, and in a number of others—in fact in a large majority of all under my care, buttermilk is better borne and more relished than sweetmilk. Clinically, my practice of allowing buttermilk, its salutary effect and the philosophy of it, is explained by recent German and French chemists, declaring that "their experiments convince them that the digestion of sweetmilk is nearly, if not quite, altogether accomplished in the small intestines." Buttermilk has less fat and casein in it than sweetmilk, hence leaves less for small bowels to do, giving them the best of all treatment, physiological rest. I am sure that too much stress cannot be placed on the quality of the milk given.

To Vaughn we are indebted for the discovery of tyrotoxicon, an alkaloid that generates in milk, and that is so poisonous. Many cases of unaccountable relapse can, I am persuaded, be traced to the use of impure milk containing this alkaloid.

To children buttermilk is not grateful; my results with the peptogenic milk powder of Fairchild are so gratifying that I bear cheerful testimony to its undoubted virtue. I am well assured that I have saved the life of several children by its use. In convalesence from this disease, whether the patient be an adult or a child, I know of no better way to hasten it, and nourish the patient, than to have the milk digested with their powder. It relieves the enfeebled digestion of much work, and thereby prolongs the rest which is so essential to all repair.

It would be an insult to this presence were I to enter into details of treatment—to mention, for instance, that the nourishment—whatever its character—should be given in small quantity and at short intervals; that bismuth in large dose would act as a sedative, absorbent and astringent; that the opium should be given for effect and not by measure; or that creosote or carbolic acid would rapidly correct the fever, etc.

To summarize and finally.

1. The disease may begin as dysentery, but therapeutically it rapidly becomes—or is complicated—with enteritis.

2. Enteric symptoms exclude mercurials, ipecac and purgatives of all kinds.

3. The failure of digestion makes medication (almost) *per orem nil.*

4. The nausea is best treated by rest—i. e., giving nothing *per orem.*

5. A superficial blister is better than a deep one.

6. While asthenia is a factor in the fatal termination, food not assimilated is an irritant.

7. The superiority of buttermilk over sweetmilk in many cases.

8. The value of peptonized foods.

